# Protocol: A multi-factorial, multi-centre study, for biomarker identification in healthy controls for comparison to babies with moderate-severe NESHIE

**DOI:** 10.1371/journal.pone.0346798

**Published:** 2026-04-08

**Authors:** Carina C. Babbo, Jeanne van Rensburg, Juanita Mellet, Sithembiso C. Velaphi, Firdose L. Nakwa, Mogomane Y.K. Masemola, Gugulabatembunamahlubi T.J. Kali, Caroline J. Foden, Solize Oosthuizen-Vosloo, Vedarsha Chellan, Odireleng Mosuwe, Priyal Mistry, Ariel R.M. Buyens, Fatima Barmania, Shakti Pillay, Daynia E. Ballot, Melantha Coetzee, Alan R. Horn, Colleen Wright, Pawel T. Schubertand, Michael S. Pepper

**Affiliations:** 1 Department of Immunology, Institute for Cellular and Molecular Medicine, University of Pretoria, Pretoria, South Africa; 2 Department of Paediatrics and Child Health, Division of Neonatology, Chris Hani Baragwanath Academic Hospital, School of Clinical Medicine, Faculty of Health Sciences, University of the Witwatersrand, Johannesburg, South Africa; 3 Department of Paediatrics and Child Health, Faculty of Health Sciences, Division of Neonatology, Kalafong Hospital, University of Pretoria, Pretoria, South Africa; 4 Department of Paediatrics and Child Health, Division of Neonatology, Tygerberg Hospital, Stellenbosch University, Cape Town, South Africa; 5 Department of Paediatrics and Child Health, Division of Neonatology, Groote Schuur Hospital, University of Cape Town, Cape Town, South Africa; 6 Department of Paediatrics and Child Health, Division of Neonatology, Charlotte Maxeke Johannesburg Academic Hospital, University of the Witwatersrand, Johannesburg, South Africa; 7 Department of Paediatrics and Child Health, Division of Neonatology, Steve Biko Academic Hospital, University of Pretoria, Pretoria, South Africa; 8 Division of Anatomical Pathology, Faculty of Medicine and Health Sciences, Stellenbosch University, Cape Town, South Africa; UNBC: University of Northern British Columbia, CANADA

## Abstract

Neonatal encephalopathy suspected to be hypoxic-ischaemic encephalopathy (NESHIE) remains a leading cause of neonatal mortality and long-term neurodevelopmental impairment, particularly in low- and middle-income countries. While therapeutic hypothermia reduces mortality in moderate to severe cases, a significant proportion of affected infants continue to experience adverse neurological outcomes. This multi-centre observational study aims to elucidate the clinical and biological mechanisms underlying NESHIE by conducting a comprehensive comparative analysis of neonates with moderate to severe NESHIE and healthy term controls. Participants with NESHIE were previously recruited under an existing approved protocol (University of Pretoria ethics reference: 481/2017), and healthy neonates will be newly enrolled. The study will integrate clinical and molecular data to: (1) identify clinical risk factors associated with NESHIE; (2) perform whole genome sequencing to detect relevant genetic variants; (3) analyse DNA methylation patterns via bisulfite sequencing; (4) assess gene expression using bulk and single-cell RNA sequencing (RNA-seq); (5) characterise proteomic and metabolomic profiles through liquid chromatography–mass spectrometry of dried blood spot samples; (6) examine the placental microbiome; and (7) evaluate placental histopathological differences between groups. By offering a multi-dimensional view of the molecular and microbial landscape of NESHIE in a South African cohort, this study aims to enhance understanding of the disease pathogenesis. Ultimately, the findings may support the development of biomarkers for early diagnosis, improve risk stratification, and guide novel therapeutic strategies for affected neonates. The study has received National Health Research Database (NHRD) registration under GP_202411_053 (Gauteng) and WC_202411_026 (Western Cape), with ethics approvals granted by the University of Pretoria (184/2024), University of the Witwatersrand (250406B), and Stellenbosch University (N24/12/154_RECIP_UP184/2024) as well as their respective tertiary academic hospitals.

## Introduction

Neonatal encephalopathy (NE) is a broad clinical condition characterized by abnormal neurological function in infants born after 35 weeks of gestation. Infants suffering from NE display altered levels of consciousness, seizures, reduced muscle tone, and breathing difficulties [1]. The aetiology of NE is diverse, including factors such as neonatal stroke, infections, metabolic abnormalities, placental abnormalities, and brain malformations [2,3]. However, in many NE cases, the aetiology remains unidentified. When NE arises following an acute perinatal event causing reduced cerebral blood flow and oxygen deprivation (hypoxia-ischaemia), it is termed hypoxic-ischaemic encephalopathy (HIE) [4].

Horn et. al. 2025 introduced the term NESHIE to describe NE due to suspected HIE, and this terminology will be applied throughout this protocol [5]. The use of NESHIE recognises that NE could be caused by mechanisms other than hypoxia; however, based on the clinical and metabolic findings available at birth, it provides the most accurate description of the condition at the time, until alternative causes can be ruled out.

Following a hypoxic-ischaemic (HI) event near the time of labour and delivery, the diagnosis of NESHIE at birth is based on a combination of clinical and laboratory findings such as low Apgar scores, acidaemia detected from umbilical cord blood (UCB) or early postnatal blood gas measurements [6–8]. Examples of an HI event include cord prolapse, uterine or placental rupture, prolonged second-stage labour, and shoulder dystocia [2,9].

The severity of NESHIE is commonly assessed using the Sarnat grading system, which categorises affected neonates into mild (stage 1), moderate (stage 2), or severe (stage 3) encephalopathy [10]. Infants with moderate to severe NESHIE are particularly at risk, with many continuing to experience cognitive difficulties that can persist into adolescence [11].

The incidence of NESHIE varies greatly between high-income countries (HICs) and low-middle income countries (LMICs), with 1–2 per 1000 live births being affected in HICs and up to 26 per 1000 live births in LMICs [11–14]. In South Africa, the incidence ranges between 2.3 and 13.3 per 1000 live births based on the health setting and defining criteria [15,16]

Therapeutic hypothermia (TH) is the only treatment known to date to reduce mortality and neurodevelopmental disability in moderate to severe NESHIE infants [18]. However, many infants undergoing TH still face neurodevelopmental challenges [11,17–19].

HIE-related brain injury occurs primarily through excitotoxicity, oxidative stress, and inflammation, leading to neuronal death by apoptosis or necrosis [20]. An understanding of the pathogenesis of NESHIE at the molecular level is critical for the development of effective treatments and preventative measures. However, potential population-specific predisposing factors/conditions that may contribute to the high prevalence of NESHIE in LMICs also need to be considered. These factors can be elucidated by investigating the molecular & clinical profiles of healthy control participants as a foundation against which samples derived from NESHIE patients can be compared. A comprehensive investigation across multiple biological levels, including genomic, epigenomic, transcriptomic, proteomic, metabolomic, and pathomicrobiomic, is therefore needed to fully characterise the context and mechanisms of NESHIE. The sections below describe the limited molecular details that are currently known about NESHIE.

### Genetics

Predisposition to NESHIE and treatment outcomes are influenced by a complex interplay of genetic and environmental factors. Recent research identified twelve candidate genes that may underlie a genetic predisposition to NESHIE: *AGT* (angiotensinogen), *AP4B1* (adaptor related protein complex 4 subunit beta1)*, CARD8* (caspase recruitment domain family member 8)*, CAT* (catalase), *COL4A1* (collagen type IV alpha 1 chain)*, IL10* (interleukin 10), *IL1B* (interleukin 1 beta), *IL6* (interleukin 6)*, MTHFR* (methylenetetrahydrofolate reductase), *NOS3* (nitric oxide synthase 3), *OLIG2* (oligodendrocyte transcription factor 2), and *TNF* (tumour necrosis factor) [21]. To date, a limited number of NESHIE-related genome-wide investigations have been conducted, leaving a significant gap in research and knowledge in this field. Evidence from genome-wide association studies suggests that the majority of disease-associated loci have been found in non-coding regions of the genome [22], underscoring the need for a whole-genome rather than a gene-focused or exome-based approach. Whole-genome analysis of 172 neonates with moderate to severe NESHIE identified 71 associated genetic variants, all located in intronic and intergenic regions, and some associated with severity or progression of the condition [23].

Identifying genetic variants associated with the diagnosis or severity of NESHIE, or the response to TH, may improve understanding of the condition’s pathogenesis, help stratify infants at higher risk, provide insights into the genetic drivers of such risk, and highlight potential prognostic markers or therapeutic targets.

It is important to ensure that genetic differences identified across groups reflect true differences between cases and controls, rather than artefacts introduced by confounding factors such as population ancestry or batch effects caused by differences in sequencing platforms or variant calling pipelines. Even small differences in the processing pipeline or underlying genetic ancestry may lead to subtle but systemic differences in allele frequencies and subsequent type I and II errors (false-positive or -negative associations). It is therefore essential to obtain control samples that match the ethnolinguistic group of the samples being studied and ensure consistency in processing and analysis.

### Epigenetics

Epigenetic changes refer to changes in gene expression that occur without alterations to the underlying DNA sequence and can be inherited. These modifications include chemical changes (e.g., methylation) to DNA or histones, as well as changes mediated by non-coding RNAs. Epigenetic changes regulate gene expression and can be influenced by environmental factors. Epigenetic studies therefore play a vital role in advancing our understanding of gene regulation and disease mechanisms. It is becoming increasingly evident that epigenetics plays a significant role in how cells respond to hypoxia. Hypoxia-inducible factor −1 alpha (HIF-1α), the master mediator of the cellular response to hypoxia, has been suggested to be under epigenetic regulation [24]. Studies have also highlighted the potential role of epigenetic programming in dictating the outcomes after hypoxic injury, such as those seen in NESHIE [25–27]. Unravelling the epigenetic landscape associated with NESHIE may provide valuable insight into the molecular pathogenesis of this disorder. Moreover, this analysis could identify biomarkers with diagnostic and prognostic value, as well as explain differences in patient outcomes.

### Transcriptomics

Transcriptomics is the study of all RNA transcripts, which includes messenger RNA (mRNA), non-coding RNA (ncRNA), and other RNA species, produced by cells at a specific time under specific conditions. By studying the transcriptome, a deeper understanding of gene expression patterns in healthy individuals can be obtained, shedding light on the normal function and regulation of gene expression. Furthermore, by looking at potential changes in gene expression patterns and biological processes, potential contributions to disease pathology can be unravelled, which will help to elucidate the underlying mechanisms [28].

The hypoxic response is a systemic adaptive process that results in the activation of a multitude of biological pathways and the regulation of gene expression. In response to hypoxia, transcription of specific genetic subsets increases significantly. Under conditions of reduced oxygen, a group of enzymes known as 2-oxoglutarate (2-OG)-dependent oxygenases are inhibited. Under normoxic conditions, these enzymes function normally, but their activity decreases in hypoxia. Prolyl-hydroxylase (PHD) is a well-studied 2-OG-dependent oxygenase, which is responsible for marking HIF for destruction [29]. HIF is a heterodimeric transcription factor composed of HIF-1α and HIF-1β. Under normoxic conditions, PHD catalyses hydroxylation of the proline residues allowing HIF-1α to bind to von Hippel-Lindau (VHL) protein. This leads to ubiquitylation and proteasomal destruction of HIF-1α [30]. However, under hypoxic conditions, suppression of PHD allows HIF to evade destruction and assemble into an active transcriptional complex. Advances in genetic and genomic analysis have shown that many genes respond to the effects of hypoxia, either directly or indirectly via HIF [31]. HIF is considered a ‘master switch’ of the molecular response to hypoxia due to its widespread hypoxia-induced expression and the diverse roles of HIF targets.

An improved understanding of the molecular mechanisms of hypoxia-induced brain injury and the associated pathologies has translational potential that could lead to a better understanding of disease pathogenesis and therefore targeted novel neuroprotective therapies [28]. The use of gene expression profiles in disease stratification needs to be explored in NESHIE. Work from the last decade has shown that hypoxia elicits unique gene expression patterns that can be measured in blood [32, 33]. Transcriptomic signatures are effectively being used in a wide range of diseases [34–36]. Current efforts are focused on developing diagnostic tests to identify babies at risk of unfavourable outcomes and identify targeted therapies for use in clinical practice [37]. This may be useful for prevention of HIE or for prediction of long-term neurodevelopmental outcome, which could aid in clinical decision making. However, since the normal birth process transiently exposes the foetus to hypoxia [38], it is important that transcriptomic profiles of clinically healthy neonates are included as relevant controls in the study of NESHIE.

### Proteomics

Proteomics is the study of the proteins and molecular mechanisms underlying the development, pathophysiology and progression of conditions such as HIE. A recent study [39] showed that haptoglobin and S100A8 proteins were upregulated in babies with NESHIE. A study by Yip et al. [40] also identified S100A8 and S100A9, which affirms the role of the S100A8/A9 complex in NESHIE, likely due to its involvement in inflammation [41,42]. In addition, proteins such as apolipoprotein D (APOD), orosomucoid 1 (ORM1), superoxide dismutase 1 (SOD1), and fatty acid binding protein 1 (FABP1) were identified as potential biomarkers for NESHIE, as their levels were significantly altered with disease severity (some upregulated, others downregulated) [43]. These proteins regulate amyloid formation, apoptosis, reactive oxygen species detoxification, and neurodegenerative pathways, all of which are relevant mechanisms of brain injury [43]. Analysis of the proteome could potentially reveal novel biomarkers associated with NESHIE when compared to the proteomes of clinically healthy control samples. This might assist in disease diagnosis, prognosis, and management [43]. In addition, monitoring proteomic changes in response to treatment could refine therapeutic strategies and assess treatment efficacy, as demonstrated in previous studies conducted on cell lines [44,45].

### Metabolomics

Metabolomics is the comprehensive analysis of small-molecule metabolites within biological systems, encompassing cells, biofluids, or tissues. Since metabolite levels fluctuate in response to physiological or pathological changes, they provide insights into the metabolic pathways that maintain normal physiological function and those altered in the disease state [46]. Therefore, metabolomics is a powerful tool for studying disease pathophysiology and identifying diagnostic and prognostic biomarkers [47]. Metabolomics is increasingly used to investigate the pathophysiology of NESHIE, with studies revealing a wide range of metabolic changes associated with NESHIE, which can be used to better understand the pathophysiology of the condition and potentially identify novel therapeutic targets [48–51].

Blood lactate, an indicator of anaerobic metabolism, has been used as a surrogate marker for tissue hypoxia or ischaemia [52,53]. While the use of lactate in evaluating the severity of NESHIE and predicting long-term neurodevelopmental outcome has to date been demonstrated in some clinical studies [54], the results have lacked sufficient statistical power to influence management. Recent advances in metabolomic profiling have enabled the identification of potential therapeutic targets and the discovery of biomarkers that could aid in treatment selection, patient stratification, and enhanced diagnosis and treatment efficacy [55]. Amongst these, several amino acids have been discovered to be significantly increased in neonates with HIE compared to healthy neonates [56]. Furthermore, glutamine decreases significantly, while the excitatory neurotransmitter glutamic acid (i.e., glutamate at physiological pH) increases significantly during HIE [49,57]. Specific acylcarnitine metabolites such as hydroxybutyrylcarnitine, tetradecanoylcarnitine, L-Palmitoylcarnitine, and more, are significantly increased in the cord blood of asphyxiated neonates who develop HIE compared to asphyxiated neonates who do not develop HIE and healthy controls. This is hypothesized to be due to impairment of mitochondrial β-oxidation of long-chain acyl-CoA under hypoxic conditions, leading to the accumulation of its precursor, such as acylcarnitines [56].

### Placental pathomicrobiome

Results from histopathology research indicate that placental infection plays a role in the pathophysiology of HIE [58]. Since the placenta is believed to be sterile, the existence of a non-infectious, commensal placental microbiome is widely debated [59]. Recent studies suggest that the placenta comprises a low-biomass microbial community, which may be altered by infection [60]. The placental microbiome has previously been linked to a range of pregnancy-related outcomes, including preterm birth and preeclampsia [61]. However, its role in NESHIE has not been extensively studied.

A clearer understanding of the placental microbiome in NESHIE may aid in determining if and how infectious agents contribute to the pathogenesis of HIE, providing insight into the severity and prognosis as well as treatment of this condition, potentially with antibiotics or other interventions. Therefore, a comparison between the placental microbiome in neonates with and without NESHIE could identify pathophysiological mechanisms and associations with NESHIE.

Taken together, an integrated analysis across multiple biological levels may provide deeper insight into NESHIE pathogenesis than any single modality alone.

Accordingly, the present study aims to compare clinical characteristics, multi-omic (genomic, epigenomic, transcriptomic, proteomic, metabolomic), and placental microbiome profiles collected from babies with moderate to severe NESHIE, as part of a previous and ongoing observational study (approval number 481/2017), to those of healthy babies recruited in the current study. Through this comparative analysis, we aim to elucidate the biological pathways underlying NESHIE and identify associated biomarkers.

## Materials and methods

### Study design

This is a multi-centre, multi-factorial, observational study that will recruit healthy babies (≥ 36 weeks gestational age) from tertiary academic hospitals in South Africa. Data obtained from these babies will be compared to existing data from a cohort of babies with moderate to severe NESHIE recruited in the ongoing observational NESHIE study (referred to as the “comparative, primary study”).

The comparative, primary study is titled: “Is there a genetic predisposition to death and disability after moderate-severe neonatal encephalopathy with suspected hypoxic ischemic encephalopathy in cooled infants? A multi-factorial, multi-centre study in a South African cohort. Amendment v. 12, Document version: 10, 21 Feb 2022”.

The ongoing comparative, primary study has received ethical approval from the following institutions:

- The Research Ethics Committee of the Faculty of Health Sciences at the University of Pretoria; approval number 481/2017,
- The Medical Research Council,
- The Human Research Ethics Committees from the Universities of Cape Town (approval number 622/2018), Stellenbosch (approval number N18/03/041_RECIP_UP-481/2017), and the Witwatersrand (approval number M180541).

### Study setting

Clinical data and samples for the healthy cohort will be collected from babies born in public hospitals in South Africa that are also participating in the comparative primary study (approval number 481/2017). Participating institutions include:

- Kalafong Hospital, University of Pretoria, Pretoria, South Africa
- Chris Hani Baragwanath Academic Hospital, University of the Witwatersrand, Johannesburg, South Africa
- Tygerberg Hospital, Stellenbosch University, Cape Town, South Africa

### Study population

The healthy cohort sub-study includes babies born at ≥ 36 weeks’ gestation who show no clinical signs of encephalopathy and no documented evidence of acute peripartum hypoxia (e.g., no sentinel events). Data from these babies will be compared to that of babies diagnosed with moderate to severe NESHIE (≥ 36 weeks’ gestation) enrolled in the comparative primary study (approval number 481/2017).

### Selection and enrolment of participants

#### Eligibility criteria for healthy cohort.

Participants will be enrolled into the study based on the following inclusion and exclusion criteria:

**Inclusion criteria for healthy cohort**

- ≥ 36 weeks’ gestation (based on EUS, foot length or Ballard)
- Birth weight ≥ 1800 g
- A 5-minute Apgar score ≥ 7
- No assisted ventilation (IPPV) required after birth
- Normal / clinically healthy newborn examination:
  - Normal blood glucose
  - Absence of clinical or serological evidence of congenital hematogenous infection (rubella, syphilis) or congenital/ suspected chromosomal abnormalities

**Exclusion criteria for healthy cohort**

- Parents who refuse consent or consent not obtained for any other reason
- Mothers who are < 18 years of age

Inclusion and exclusion criteria for the moderate and severe NESHIE babies enrolled in the comparative primary study are listed in S1 Annexure.

### Informed consent process

Research team members will collect UCB and placental tissue from eligible newborns. Eligibility will be determined according to predefined inclusion and exclusion criteria. Maternal and infant clinical data will be recorded using standardised data capture forms (see S3 Annexure in S3 File). All study personnel will be trained and certified in Good Clinical Practice (GCP) prior to the initiation of recruitment. When consent for study participation is obtained, additional consent for infant blood sampling and genetic testing will also be requested (see S2 Annexure in S2 File).

While the core procedures will be consistent across sites, there are minor differences in the recruitment approach. At Chris Hani Baragwanath Academic Hospital, research nurses will approach mothers after delivery. Attending obstetricians will be requested to delay discarding the umbilical cord and placenta of potential candidates until a research nurse has had the opportunity to speak with the parent(s) and obtain informed consent. Consent will not be sought from mothers who are in active labour or under the influence of sedatives. Potential candidates include mothers undergoing booked caesarean sections, planned inductions, or uncomplicated spontaneous vaginal deliveries. If the parent(s) decline participation or cannot be contacted within 24 hours of birth, the samples will be safely discarded.

At the remaining sites, Kalafong Provincial Tertiary Hospital and Tygerberg Hospital, a research team member will be based at the antenatal care (ANC) clinic. Mothers in their third trimester will be approached, informed about the study, and asked to provide pre-consent to allow for re-contact at the time of delivery (see S2 Annexure in S2 File). A note will then be made in the mother’s file to notify the obstetrics team that she is a potential candidate for the study. Upon hospital admission for delivery, the research team member will request that the attending obstetricians delay discarding the umbilical cord and placenta until informed consent is obtained. The final consent should be obtained within 24 hours of birth. Once again, no consent will be sought during active labour. If consent is not obtained or if the parent(s) cannot be re-contacted within the specified timeframe, the samples will be safely discarded.

The consent documents used in this study are provided in S2 Annexure in S2 File. Please note that these documents were developed for use in South African public sector hospitals serving linguistically and socioeconomically diverse populations, where English is frequently not a first language. During ethics review, we were specifically instructed to prioritise plain, direct language to optimise comprehension across varying literacy levels and to avoid technical terminology that may be abstract or unclear in this setting.

### Recruitment

NESHIE occurs exclusively in babies; therefore, all study participants will be babies. The majority of patients attending the participating public hospitals are from low- to middle-income backgrounds and are demographically representative of the South African population. There will be no preference for gender selection.

Participants will be recruited within 24 hours of birth. This time frame has been established due to the fact that healthy babies and mothers are often discharged quickly, should no complications arise. The babies will be assessed using the data capture sheets designed for this purpose (S3 Annexure in S3 File) to determine whether they meet the study’s inclusion criteria. Infants who meet the specified inclusion criteria will be eligible for screening. Following this process, those without any exclusion criteria will be enrolled if their parent(s) provide consent.

Enrolment into the study requires the fulfilment of all three of the following conditions: (1) all inclusion criteria must be met; (2) no exclusion criteria may be present; and (3) informed consent for participation must be obtained. No data or samples will be retained from infants who do not meet the inclusion criteria. However, for infants who meet the inclusion criteria but are excluded due to the presence of exclusion criteria or the lack of informed consent, limited demographic information and the reason for exclusion will be documented to characterise the screened population (S3 Annexure in S3 File).

### Sample size

NESHIE study sites are located in the Gauteng and Western Cape provinces of South Africa. According to the Stats SA report for 2021, a total of 203 248 and 93 076 live births were registered in each province, respectively (Statistics South Africa, 2021).

Based on a sample size estimation for an unmatched design with a two-sided confidence level (alpha = 0.05), 80% study power, and a control-to-case ratio of 499:1, the Fleiss method with a continuity correction requires a minimum sample size of 304 participants. This assumes that 0.1% of the controls and 99.9% of the cases will have been exposed to a pathogenic hypoxic event. As shown in the S5 Annexure in S5 File, these estimates were calculated using the OpenEpi software (https://www.openepi.com). However, in order to match the number of NESHIE samples obtained in the primary comparative study, it is estimated that a total of 350 healthy participants need to be enrolled.

### Schedule of events

The samples that will be collected are shown in Table 1. The collection of these samples will not cause any discomfort to the baby or mother. The placenta and cord blood are routinely disposed of as biological waste.

**Table 1:** Sample collection from healthy babies.

Sample	At birth	0-6 Hrs
Whole Placenta^a^	X	
Placenta Biopsy	X	
Dried Blood Spots^b^		X
Umbilical Cord Blood	X	
Blood Gas	X	

*^a^– Whole placentas will only be collected from Chris Hani Baragwanath Hospital*

*^b^– Umbilical cord blood will be used for the dried blood spot cards*

The clinical examinations that will be performed are listed in Table 2 below.

**Table 2:** Neonatal examinations.

Exam	At birth	0-6 Hrs
Thompson Score ^b^		X
Sarnat Grade ^b^		X
APGAR Score	X	

*^b^– To be performed if possible, within 6 hours of birth*

## Methods

While the service providers, analytical processes, and pipelines described below reflect current study plans, modifications may be implemented if deemed necessary by the study team and approved by associated Research Ethics Committees, provided such changes do not materially affect the integrity or objectives of the planned analyses.

### Patient management

Apart from non-standard blood sampling from UCB for multi-omics assessment and neurological investigations, patient management will not deviate from standard clinical practice. This study is therefore observational and investigational in nature. A general and neurological examination (including a Modified Sarnat Grade [8,62] and Thompson HIE score [63]) will be done by the attending medical staff around the time of birth (0–6 hours), if possible.

### Genomics, epigenetics, and transcriptomics

#### Sample collection.

Umbilical cord blood (UCB) collected within 60 minutes of birth will be transferred into a tube containing a nucleic acid (DNA/RNA) stabilization agent to preserve nucleic acid integrity for downstream processing and analysis.

To collect the UCB, clamp the cord, and clean the area where the needle will be inserted. Carefully insert the needle into the umbilical vein, ensuring that blood flows into the collection line. Transfer the collected blood into a blood collection tube (up to 3 mL) using a Vacutainer® system. Once the required volume has been obtained, clamp the collection line, remove the needle, and dispose of it according to standard hospital procedures. Gently invert the blood collection tube 10 times to mix, and store and transport at ambient temperature (4–25 °C).

This sample collection strategy will apply to the use of UCB samples for genomics, epigenomics, and transcriptomics components of this investigation.

#### Total DNA extraction.

Total DNA will be extracted by Inqaba Biotec from nucleated cells using the Quick-DNA^TM^ Miniprep Plus Kit (Zymo Research). Isolation strategies and kits may be adjusted and optimised as needed. The quantity, quality, and purity will be measured using standard equipment. In addition, DNA integrity and quality will be assessed using a TapeStation 2200 (Agilent Technologies). This extraction method will be applied for the isolation of DNA for the purposes of genomic and epigenomic investigations.

#### Total RNA extraction.

Total RNA from nucleated cells will be extracted by Inqaba Biotec using the New England Biolabs RNA extraction protocol, or equivalent kit and optimised protocol. The quantity, quality, and purity of the RNA for RNA-seq will be measured using standard equipment (e.g., Nanodrop®). Additionally, RNA integrity and quality will be assessed using a TapeStation 2200 (Agilent Technologies). This extraction method will be used to isolate RNA for the purposes of transcriptomic investigations.

#### Whole-genome sequencing: Genomics.

All DNA samples will preferentially undergo whole genome sequencing using either Illumina® or BGI-based sequencing platforms (whole exome sequencing may also be performed). The required quantity of DNA will be shipped to the appropriate sequencing institution/centre. Once the sequencing data has been returned (as *.fastq files via a secure electronic channel), they will be trimmed to remove all adapter sequences, and variant calling will be performed.

#### Data analysis: Genomics.

The allele frequencies of genetic variants, whether contained within a set of genes implicated in NESHIE or within the entire genome, will be compared. The comparison will include control (healthy babies) and patient (babies with moderate/severe NESHIE) whole genome sequencing datasets. Analysis will be guided by the nature of the data and may include collapsing variants into genes or genomic regions, comparing them to global allele frequencies, and using variant effect prediction metrics to determine whether there are statistically significant differences between variants in healthy babies compared to babies diagnosed with moderate and severe NESHIE. Fisher’s Exact test will be used to determine if there are statistically significant differences in genetic variant allele frequencies between moderate/severe NESHIE babies and healthy babies. A suitable significance threshold will be used, depending on the dataset, with appropriate correction for multiple testing applied. Collapsing methods to test the aggregate effects of variants within genes will be performed using the sequence kernel optimisation test – optimised (SKAT-O) with appropriate multiple testing correction.

#### Bisulfite treatment and sequencing: Epigenetics.

Bisulfite sequencing is a technique used to analyse DNA methylation patterns by detecting methylated cytosines in genomic DNA at single-base pair resolution. Extracted genomic DNA is treated with sodium bisulfite, which chemically converts unmethylated cytosine residues to uracil through deamination, while methylated cytosines remain unaffected. Following bisulfite treatment, DNA is desulfonated (to remove the bisulfite reagent), purified (to remove residual salts), and denatured into single-stranded DNA. The single-stranded DNA is then amplified by polymerase chain reaction (PCR) to create sequencing libraries. Upon sequencing, methylated cytosines are read as cytosines, while unmethylated cytosines are read as thymines. By comparing bisulfite-treated and untreated sequences, the location of the methylated cytosines can be determined. Bisulfite treatment, PCR, library preparation, sequencing, and data analysis will be performed in collaboration with Human Immune Monitoring Centre (HIMC), Stanford University, or equivalent collaborator or service provider.

#### RNA-sequencing: Transcriptomics.

Purified RNA will be converted to complementary DNA (cDNA), which will be used to create sequencing libraries to enable analysis of coding and multiple non-coding RNA forms. Sequencing libraries are generated through the fragmentation of cDNA and the attachment of adapters to both ends of the resulting fragments, facilitating their attachment to the flow cell of the sequencer. Fragments will then be amplified and purified. Barcoding occurs during amplification and enables the pooling and sequencing of multiple samples in the same reaction. Library preparation and sequencing will be performed by the HIMC at Stanford University (USA) through the Global Health Discovery Collaboratory or the SAMRC Genomics Centre in Cape Town, South Africa, using BGISEQ, MGISEQ or equivalent sequencers. A read depth of up to 50 million paired-end reads per sample will be generated to obtain coverage over the full transcript libraries, including rare and poorly expressed transcripts.

Initial experiments will determine gene expression through bulk RNA analysis; however, it may be necessary and more informative to analyse gene expression at a single-cell level at a later stage. The reason is that when detecting gene expression in populations, small alterations might go undetected when analysing bulk RNA. Single-cell gene expression will enable the detection of gene expression changes in less abundant cell populations that would otherwise be masked by the gene expression of other cells within a given population.

#### Data analysis: Transcriptomics.

FASTQ format files containing raw sequencing data generated by RNA-seq platforms will be used in the bcbio-nextgen workflow (https://github.com/chapmanb/bcbio-nextgen) to assess the quality of the data, align sequencing reads to the human reference genome (GRCh38), and perform gene and transcript quantification. The bcbio workflow enables both gene- and transcript-level quantification. A MultiQC report will be generated and will provide information on data quality (Phred scores), read count per sample, and percentage alignment to the human reference genome (GRCh38). Aligned reads will be converted into summarised gene counts and normalised for gene length. Genes significantly differentially expressed between moderate-severe NESHIE and healthy control babies will be identified.

Statistical analysis will be performed using R (R Project for Statistical Computing) packages. The identified set of differentially expressed genes will then be correlated to the clinical parameters in these babies and subjected to pathway analysis (Ingenuity Pathway Analysis) to identify enriched biological pathways. A p-value of < 0.05 will be considered significant. Statistical analysis approaches will also be required for this study; however, the exact approach will be informed by the data at the time of data analysis. Analyses for this study will be run using the high-performance computing cluster of the Institute for Cellular and Molecular Medicine (ICMM) and the Centre for Bioinformatics and Computational Biology at the University of Pretoria in collaboration with the HIMC, Stanford University.

### Proteomics and metabolomics

#### Sample collection.

A total of 75–80 µL of UCB will be spotted onto dry blood spot (DBS) cards at 0–6 hours after birth. The blood drops should sufficiently fill and saturate the circles indicated on the DBS card. No repeat blood spot applications should be performed within a single circle. A total of four to five large blood spots will be collected from each baby on a single blood spot card.

Following sample collection, the DBS cards should be thoroughly dried for at least 3 hours on a horizontal surface ensuring that the collection area does not come into contact with any other surface. The cards should be protected from direct sunlight, and excessive heat and/or moisture. Once the blood spot cards are completely dry, they should be safely stored at room temperature in the supplied sample storage envelopes until collected/transported to the designated laboratory for further analysis

Of the four to five large blood spots collected on the DBS card, one to two will be used for proteomics analysis.

#### Sample processing: Proteomics.

Proteomics analysis will be performed by Precision Biomarker Laboratories (PBL) in Los Angeles, California, USA (or equivalent service provider). The protein content in DBS will be extracted and digested into peptides. Peptides will be cleaned up and subjected to liquid chromatography (LC) separation by running a 60 min gradient of organic solvent to elute peptides in increasing hydrophobicity order, before mass spectrometry (MS) analysis. Analysis will be performed on a Thermo UltiMate 3000 LC system coupled to a Thermo Orbitrap Exploris 480. MS data on the Orbitrap will be collected with a data-independent acquisition (DIA) method collecting peptide precursor and fragmentation information. DIA MS raw files will be analysed using ProEpic in-house proprietary omics software/bioinformatics platform. The raw files will be converted to mzML(txt) format, and the raw intensity data for peptide fragments will be extracted from DIA files using the OpenSWATH workflow and searched against the Human Plasma spectral library.

#### Data analysis: Proteomics.

Proteins present in the DBS samples of NESHIE babies will be compared to the proteins present in the DBS samples of healthy controls. Among others, covariates such as sex, blood pH and base excess, Thompson HIE scores, and Modified Sarnat grades will be included in the analysis. The results will be summarised, and the findings will be represented visually to help identify the biological patterns and trends.

#### Sample processing: Metabolomics.

Metabolomics analysis will be performed by Sapient Bioanalytics in San Diego, California, USA, or an equivalent facility. Samples will be processed, and metabolites extracted for analysis. The samples will then be run on an optimised high-throughput LC-MSy-based metabolomics system for non-targeted measures of thousands of small molecules. Internal standards are spiked into each sample as well as pooled replicate samples which are run in regular intervals and simultaneously analysed for quality control / quality assurance. The completed metabolomics dataset is then extracted and processed. Raw mass spectral data files, and a dataset description file including QC/QA information will be generated.

The chemical identity of the metabolites measured in the dried blood spots is not always known before experiments are initiated. It might therefore become necessary to identify the most significant metabolites. Signals will be identified using commercial standards and MS/MS spectra will be compared to reference spectra. Metabolite mass, retention time, and MS/MS fragmentation patterns will be searched against Sapient Bioanalytics’ in-house library of over 800 commercial standards and public repositories of MS/MS data.

#### Data analysis: Metabolomics.

Data analysis will be performed using Sapient Bioanalytics’ in-house R scripts and specific software packages. Statistical analysis will be performed using R and will include regression analyses to determine if molecules associate with clinical parameters. Advanced bioinformatics and systems biology approaches will be used to integrate metabolomics data with other omics datasets (including genomics, epigenomics, transcriptomics, and proteomics) to construct a multi-omics framework.

### Placental microbiome and histology

#### Sample collection.

Prior to formalin preservation of the placentas, a tissue biopsy sample should be obtained within a 2 cm radius of the cord insertion point by using a 2 mm tissue biopsy punch or scalpel set. Care will be taken to avoid piercing the main veins in this region. No more than 100 mg of tissue should be collected. Collected samples will immediately be placed into a Zymo DNA/RNA Shield tissue lysis tube with 3 mm bashing beads (Irvine, CA, USA). The lysis tube will be vortexed for 10–15 seconds to fully homogenise the tissue. The collected sample should be stored at ambient temperature (4–25°C) until shipped to Wits Diagnostic Innovation Hub (Wits DIH; Braamfontein, Johannesburg, South Africa) (formerly known as Clinical Laboratory Service – CLS), a Division of the Wits Health Consortium, for storage. At Wits DIH, samples will be briefly vortexed again and stored at −80ºC.

As soon as possible following tissue biopsy collection, the placenta must be placed in a bucket containing sufficient 10% buffered formalin to cover the specimen and allow adequate fixation. The entire placenta must be sent to the laboratory and stored at room temperature for 24–48 hours. Histological examination of placentas will be performed by dedicated study-associated placental pathologists through the National Health Laboratory Services.

#### Sample processing and analysis of tissue biopsy samples.

Nucleic acid isolations will be performed at the Centre for Microbial and Ecological Genetics (CMEG) at the University of Pretoria, Pretoria, South Africa. Samples will be shipped from Wits DIH and thawed. The QIAamp DNA Mini Kit (or equivalent isolation kit) will be used to extract nucleic acids following the manufacturer’s protocol, with optimization for the detection of low-biomass microbial species. Extracted nucleic acids will be quantified and quality-checked using NanoDrop or Qubit assessments. DNA and RNA integrity (DIN and RIN) assessments will be performed as needed using a Tapestation.

For sequencing and analysis, samples will preferentially be prepped for shotgun metagenomics analysis through the Centre for Proteomic and Genomic Research (CPGR; Cape Town, South Africa) or an equivalent service provider. Alternatively, 16s ribosomal RNA (rRNA) amplicon sequencing of the V6-V8 region will be performed. CPGR would be responsible for the sample preparation, including but not limited to sample clean-up, rRNA depletion (if necessary), and cDNA synthesis. Sequencing will preferentially be performed using an Illumina MiSeq or NovaSeq 6000 platform. Sequences will be filtered, quality-controlled, and analysed with a bioinformatics pipeline in collaboration with CMEG. This analysis will include diversity assessment, comparative meta-analysis, and correlation analysis. Raw data will be received in FASTQ format and annotated using SILVA or an equivalent database.

#### Sample processing and analysis of histology samples.

The fixed placentas will be sampled according to standard protocol: 1 cassette containing 2 sections of umbilical cord – proximal and distal – and membrane roll; 4 cassettes containing parenchyma, including maternal and fetal surface as well as any relevant macroscopic pathology identified. These cassettes will be processed into paraffin-embedded blocks according to standard protocols. Haematoxylin and Eosin-stained slides will be produced from the paraffin-embedded blocks using standard procedures. The placental slides will then be microscopically examined, and results reported using a standard template.

If cytomegalovirus (CMV) or mycobacterial infection is suspected based on the observed pathological features, Ziehl–Neelsen staining for mycobacteria and immunohistochemistry for CMV and mycobacteria will be performed on the placental tissue. The remaining placental tissue in formalin will be stored for 6 months.

### Data Handling

#### Data collection and storage.

To ensure participant privacy and data security, all collected data will be anonymised. Each participant will be assigned an alphanumeric code, that replaces any personally identifiable information. This code will allow researchers to accurately correlate genotypic data with the corresponding clinical information while maintaining confidentiality. The code structure will indicate the study site from which the sample originated, enabling proper classification and analysis. No direct identifiers, such as names or dates of birth, will be linked to the research dataset, and only authorized personnel will have access to the information that connects codes to participant identities, ensuring compliance with ethical and regulatory standards.

Phenotypic data will be collected via data capture sheets (S3 Annexure in S3 File). Data will be collected by appropriately qualified and trained research team members and will be entered into a REDCap database. Data will be subjected to quality control checks to assist with identifying errors in data entry. Each study participant’s record will be linked to an electronic copy of the data capture sheet and a barcode that indicates the location of the associated samples in the bio-repository. Treatment strategies and associated rationale, as decided by medical staff, will also be captured.

Data generated during the execution of the bioinformatics protocols will be stored on a high-performance cluster, or equivalent UP-based server at the Institute for Cellular and Molecular Medicine (ICMM). All data will also be stored on the ICMM’s high-performance computing cluster, which will power the bioinformatics analyses. The cluster has 320 TB of storage, 20 cores and 128 GB of RAM dedicated to computation and a memory node with eight cores and 256 GB of RAM. The server is protected by a firewall that does not allow the creation of open ports. Access to the server from outside the University of Pretoria requires the creation of a secure tunnel using VPN software. An equivalent system hosted at the Department of Bioinformatics at the University of Pretoria’s Hatfield campus may also be used for the storage and analysis of genomic data. Analysis may also be performed via equivalent bioinformatics infrastructure facilitated by collaborators should the computational burden of the analyses on UP infrastructure be too severe. This would be facilitated with the use of a VPN and on condition that any data analysed through our collaborators’ infrastructure be removed in its entirety upon completion of the analyses.

Data generated as part of the collaboration with the Human Immune Monitoring Centre (HMIC), Stanford University, who will also assist us with the computational analysis, will only be used for the purposes of this study. This will be stipulated in the material (sample and data) transfer agreement (MTA).

Data generated by our service providers will be retained by them as a backup until the study has been completed. The data will be exclusively used for this study, as specified in the MTA.

#### Data management plan.

This sub-study will follow the same data management plan (DMP) as the NESHIE observational study, i.e., the primary study (481/2017) (Strydom, Van Rensburg, & Pepper, 2023). The data management plan describes the ethical governance of data generated through standard clinical practice, as well as that generated through the analysis of a variety of samples collected from research participants and analysed using multi-omics approaches. The following core elements are discussed in the DMP:

a. Project and Sample/Data Collection – describes the management of the project and the sample/data collection process
  - Research team and training
  - Purpose and strategy
  - Regulatory requirements and informed consent
  - QA and QC processes
  - Ownership
  - Auditing
  - Website
b. Data Characteristics – describes the data collected or generated during the research
  - Data collection sheets
  - Data submission
  - Sensitive and confidential data
  - Data identifiability
  - Data updates
  - Data reporting
c. Data Storage and Security – describes the processes implemented to store the data in a safe and secure manner
  - Database design, creation and maintenanc
  - Data input and processin
  - Data format and transformation
  - Data standard and metadata
  - Data storage and database security
d. Sample/Data Access and Sharing – describes access control and sharing of the sample/data for future use or re-use
  - Sample/data sharing intentions and access requirements
e. Data Archiving – describes the long-term storage requirements of the data after the completion of the project to ensure all stakeholder compliance
  - Retention period
  - Repositories
  - Future use of data and Data Access Committee (DAC)

#### Data monitoring and validation.

Site investigators will manually enter data from the case report forms (CRFs) in S3 Annexure in S3 File for all screened participants into electronic forms within REDCap. Participants who fail to meet the inclusion criteria or who meet any exclusion criteria will have limited demographic information recorded, along with detailed documentation of the inclusion and exclusion criteria, during screening. Enrolled participants will have all relevant CRFs uploaded onto REDCap. This includes screening & enrolment information, sample collection forms, and neonatal and maternal CRFs. Informed consent forms, which contain identifying information, will be exclusively uploaded to LogicalDOC; a secure document repository.

The enrolment process is expected to be dynamic across all the study sites and will involve extensive data collection from both the baby and mother (see S3 Annexure in S3 File). To efficiently manage this data, automated alerts will be configured within REDCap. These alerts trigger email notifications upon data entry events such as the recruitment of a new participant or the uploading of reports.

As data uploads become available, the information will be reviewed alongside previously captured data. REDCap’s record locking feature allows forms to be secured by “locking” them after thorough review and verification. When data inconsistencies are identified, queries will be directed to the relevant investigators via the REDCap Workflow Resolution system. Once queries are resolved and necessary adjustments are made, the corrected CRFs will be uploaded to the designated Upload section, and the form will be locked.

REDCap also incorporates data validation rules that can be configured to identify errors, inconsistencies, or missing data within the captured data. For instance, an alert can be triggered if a recorded data point falls outside a specified range for laboratory results or if required data fields are incomplete. Additionally, a comprehensive audit trail will be maintained through weekly downloads of logging details from both REDCap and LogicalDOC.

#### Ethical considerations and declarations.

This study involves human participants and was conducted in accordance with the ethical principles outlined in the Declaration of Helsinki and follows good clinical practice guidelines.

Ethical approval for the study was obtained from the following institutional research ethics committees:

- University of Pretoria (Reference: 184/2024)
- University of the Witwatersrand (Reference: 250406B)
- Stellenbosch University (Reference: N24/12/154_RECIP_UP184/2024)

Approval was also obtained from the respective hospitals, and the study was registered with the NHRD under GP_202411_053 (Gauteng) and WC_202411_026 (Western Cape).

Written informed consent will be obtained from all participants’ parents prior to any study-related procedures. The consent process includes detailed explanations of the study aims, procedures, potential risks and benefits, data handling practices, and the voluntary nature of participation. Parents will be informed that participation could be withdrawn at any time without any adverse consequences.

Only trained personnel will obtain consent, and extra care will be taken to ensure comprehension by caregivers. The study’s focus on NE necessitates the inclusion of babies to better understand the condition and identify potential biomarkers for early diagnosis and intervention.

All clinical data is anonymised and stored in a secure, access-restricted NESHIE database. Personal identifiers are removed to maintain confidentiality. Only authorised members of the research team have access to the de-identified data. Data used for analysis and publication will remain anonymised. Some anonymised omic data (e.g., transcriptomic, proteomic, metabolomic, and microbiomic) may be shared with approved data repositories. However, genomic and epigenomic data will not be deposited into public repositories in accordance with restrictions set by the University of Pretoria’s Faculty of Health Sciences Research Ethics Committee, due to the vulnerability of participants, consent through proxy, and the high potential for re-identification.

This study poses minimal risk to participants, primarily limited to samples that would usually be discarded. There are no direct benefits to individual participants; however, the study aims to contribute to improved understanding and clinical management of NESHIE in future populations.

### Status and timelines

Participant enrolment commenced on 01/06/2025 and is expected to continue until the targeted enrolment number is obtained, which is envisaged for 31/03/2026. End of enrolment will also mark the end of data collection as there are no follow-up visits. Following the completion of enrolment, all clinical data will undergo validation and quality control procedures. Once data verification is complete, biological samples will be processed and shipped for omic and histological analyses. The anticipated timeline for obtaining results will depend on the completion of sample analyses. These analyses are subject to the availability of funding, and will be initiated once funding has been secured. Subsequent phases will include data analysis and interpretation.

## Discussion

This multi-centre, multi-omic observational study aims to generate a comprehensive biological reference profile of healthy term babies. By establishing a baseline dataset across genomic, epigenomic, transcriptomic, proteomic, metabolomic, and microbiomic domains, this study aims to gain a better understanding of disease pathogenesis and identify biomarkers for early detection and improved clinical management. In order to achieve this goal, the datasets generated will be compared with those from babies diagnosed with moderate to severe NESHIE in the ongoing comparative primary study.

A strength of this study is that healthy control participants are being recruited from the same clinical setting as the previously enrolled moderate to severe NESHIE cohort. This approach ensures that both groups share similar sociodemographic, environmental, and healthcare system exposures, thereby reducing the potential for confounding factors. Furthermore, it ensures uniformity in sample collection, processing, and storage procedures. These factors strengthen the validity of downstream multi-omic, histological, and microbiomic comparisons, allowing for more accurate identification of disease-specific biological signatures.

Results from the study will be disseminated through peer-reviewed publications and conference presentations. We also aim to engage with South African clinicians and scientists to ensure that findings contribute meaningfully to local neonatal care strategies.

## Supporting information

S1 File Annexure 1:Inclusion and Exclusion Criteria for Moderate and Severe NESHIE Babies in Primary Study. Enrolment criteria used to determine participant eligibility for the primary NESHIE study.
(PDF)

S2 File Annexure 2: Informed Consent Documentation. All documentation related to informed consent, including recontact forms, consent forms, participant information leaflets, consent checklist and processes for the study.
(PDF)

S3 File Annexure 3: Data Capture Forms. Standardised data capture forms used to record maternal and infant clinical data.
(PDF)

S4 File Annexure 4: Community Engagement Form. Document used to guide and record community engagement activities related to the study.
(PDF)

S5 File Annexure 5: Sample Size Calculation. Details of sample size calculations performed for the study.
(PDF)
