# Supplementary material for: Protocol: A multi-factorial, multi-centre study, for biomarker identification in healthy controls for comparison to babies with moderate-severe NESHIE
Source: PLoS One. 2026 Apr 8;21(4):e0346798. doi: 10.1371/journal.pone.0346798 (PMC13061247; doi:10.1371/journal.pone.0346798)
Supplement: S1 File — Enrolment criteria used to determine participant eligibility for the primary NESHIE study. (PDF) [file pone.0346798.s001.pdf]

Annexure 1: Inclusion and Exclusion Criteria for Moderate and Severe NESHIE Babies in Primary Study

The inclusion and exclusion criteria from the primary study "***Is there a genetic predisposition to death and disability after moderate-severe neonatal encephalopathy with suspected hypoxic ischemic encephalopathy in cooled infants? A multi-factorial, multi-center study in a South African cohort.*** **Amendment v. 12, Document version: 10, 21 Feb 2022"** is shown below.

Moderate/Severe NESHIE Inclusion Criteria

**All of the following:**
1. ≥ 36 weeks gestation (based on EUS, foot length or Ballard)
2. Birth weight ≥ 1800g
**AND** Suspected intrapartum hypoxia based on the presence of at least **one** of the following:
1. A blood gas (umbilical cord or any infant blood) within 60 minutes of birth showing acidosis with a pH of ≤7 or base deficit ≥ 16 mmol/l; **or**
2. A blood gas (umbilical cord or any infant blood) within 60 minutes of birth showing acidosis with a pH of ≤7.15 or base deficit ≥ 10 mmol/l with a history of a perinatal event (eg: sudden onset bradycardia, cord prolapse/rupture, uterine rupture, shoulder dystocia, maternal trauma/haemorrhage/arrest; **or**
3. A 5-minute Apgar score of < 7 (only if early gas is not available)
4. A need for any type of resuscitation/assisted ventilation at 10 minutes.
**AND** Moderate-severe neonatal encephalopathy based on all of the following:

1. Clinical signs of encephalopathy (lethargy, stupor, coma, seizures or Thompson score ≥ 7) and, if seizures are absent, one or more of hypotonia, abnormal reflexes or an abnormal suck;

Moderate/Severe NESHIE Exclusion Criteria

1. Parents who refuse consent;
2. Mothers who have not been counselled by a medical officer prior to being approached for informed consent
3. Infants who are too sick or unstable to cool: uncontrolled bleeding, systemic hypotension, and/or pulmonary hypertension (FiO2 > 0.8) that do not respond to treatment;
4. Infants who meet resuscitation *discontinuation* criteria during the first 10 minutes of life (asystole)
5. Congenital/ suspected chromosomal abnormalities expected to affect intellectual or motor outcome;
6. Clinical or serological evidence of congenital hematogenous infection (rubella, syphilis);
7. Surgical abnormalities expected to interfere with cooling;
8. Infants who are moribund with agonal breathing or persistently absent respiratory effort
9. Infants who die before consent is obtained
10. Consent not obtained for reasons not already indicated (i.e. reasons beyond parents refusing consent, and infants who die before consent is obtained)
11. Infants in which the primary physician discontinues cooling before 72 hours of treatment for clinical reasons other than for prognostic purposes alone
12. The neonate was not cooled before 6 hours of life
13. The neonate was not cooled
14. Mothers who are <18 years of age
