# Supplementary material for: Protocol: A multi-factorial, multi-centre study, for biomarker identification in healthy controls for comparison to babies with moderate-severe NESHIE
Source: PLoS One. 2026 Apr 8;21(4):e0346798. doi: 10.1371/journal.pone.0346798 (PMC13061247; doi:10.1371/journal.pone.0346798)
Supplement: S2 File — All documentation related to informed consent, including recontact forms, consent forms, participant information leaflets, consent checklist and processes for the study. (PDF) [file pone.0346798.s002.pdf]

Annexure 2.1: Informed Consent for Recontact Healthy Controls

May we contact you about our research study?

Who are we?
Hello, my name is [RESEARCH NAME HERE], and I am one of the doctors or research team members working at [HOSPITAL NAME HERE] and [ASSOCIATED UNIVERSITY NAME HERE].

What is the background and why are we talking to you today?
When we do research to understand sickness in babies better, it is often necessary for scientists to collect information and samples from both healthy and sick babies. This helps us fully understand how sick babies differ from healthy babies, and often guides us in finding ways to better treat (and sometimes cure) sick babies.

Data & Sample collection
After you give birth, the doctor will discard your placenta, the umbilical cord and the umbilical cord blood. If you choose to join our study, we would like to ask the doctor to collect your placenta and umbilical cord blood for our research study.

The collection of these samples will have no effect on you or your baby, and will not cause any extra discomfort or pain to you or your baby. If we collect these samples, they will be stored appropriately in a locked room and no-one will use them for this study unless you give us permission to do so.

If we collect the samples and information, we will ask you if we may include them in the research study. We would collect information that doctors use to understand your health before pregnancy, during pregnancy, and during the birth process. Information on the health of your baby during and after birth would also be collected.

If we collect the sample and information at birth but don’t get to speak to you again for any reason, we will not use the samples and will dispose of them. We will respect your choice to participate and not use any collected samples or information unless you give us permission to include them in our research study.

If you have any questions, we can discuss them now. You are also welcome to call if there is anything else you would like to know later. If you have any additional questions please call:

Name: ____

Phone number: ____

There are some questions that we would like you to please answer. This includes providing your contact information. By answering these questions and signing the form, you are giving us permission to have and use this information as part of this research project. We will not share this information with people outside of the study. Only a small number of people who are part of the research team will be able to see this information. Please complete the next page.

Your name & surname: ______________________________
Cell/Tel: ______________________________
Email: ______________________________

Alternative contact person
Relationship: ______________________________
Name & surname: ______________________________
Cell/Tel: ______________________________

- I agree that you can contact me again once I have given birth in order to obtain my informed consent to participate in the research study.

  ☐ **YES** ☐ **NO**

- I agree that you can store the samples that have been described here from myself and my baby only until we have spoken again about your study and I have decided if I want to join it.

  ☐ **YES** ☐ **NO**

Signature: ______________________________
Signature date: ______________________________

Study ID

**AUTHORISED PERSONNEL TO COMPLETE**	
Mother hospital number:	
Name & surname of person performing recontact -consent:	
Signature:	
Signature date:	
Was a signed copy of this document given to the parent(s)	Yes / No

Annexure 2.2: Participant Information Leaflet & Informed Consent for Study Participation of Healthy Controls

STUDY TITLE:

A multi-factorial, multi-centre study, for biomarker identification in healthy controls for comparison to babies with moderate-severe NESHIE

STUDY NUMBER:

**National Health Number GP:**	GP_202411_053
**National Health Number WC:**	WC_202411_026
**Ethics approval number UP:**	184/2024
**Ethics approval number WITS:**	250406B
**Ethics approval number SU:**	N24/12/154_RECIP_UP184/2024

PROJECT FUNDED BY: South African Medical Research Council
Bill and Melinda Gates Foundation

NATIONAL PRINCIPAL INVESTIGATOR: Professor Michael Pepper (University of Pretoria)

LOCAL PRINCIPAL INVESTIGATORS: Dr Magomane Masemola (University of Pretoria)
Prof. Sithembiso Velaphi (University of the Witwatersrand)
Dr Gugu Kali (Stellenbosch University)

INSTITUTIONS INVOLVED: University of Pretoria,
University of the Witwatersrand,
Stellenbosch University

DATE AND TIME OF FIRST INFORMED CONSENT DISCUSSION:

**Day**	**Month**	**Year**

:
**Time**

PARTICIPANT INFORMATION LEAFLET

Hello, my name is ___[Research Member Name Here]___, and I'm part of the research team at ___[Hospital Name Here]___ and the ___[University Name Here]___. We're conducting a study called the NESHIE study, and we'd like to collect information and samples from you and your baby.

The goal of the study is to better understand why some babies are born healthy (like your baby) and others are born sick with brain injury from low oxygen levels. **The information about this study has been provided in a separate leaflet**. The study involves gathering information and samples from you and your baby and comparing them with information from babies who are sick. The study does not offer any extra treatment or care compared to what is normally provided.

We're inviting you and your baby to take part in the study. You won't be paid, and it won't change how doctors care for you or your baby. However, the study might help sick babies in the future. If you agree, we will collect information from your medical records and your baby's medical records, including details about your pregnancy and birth. As explained, we will also collect some blood, blood spots, and placenta samples.

This consent form may have some words you don't understand. Please ask the study doctor or staff to explain anything that is unclear. You can talk to your family or friends before deciding to take part in the study. You should not agree to join the study unless you understand what it's about and what's expected of you and your baby.

You do not have to say yes, and you can say no. It's your choice.

If you agree now, you can change your mind later, without needing to explain why.
If you choose to take part in this study, or not, it will have no effect on the medical care you or your baby receive. You and your baby will continue to receive treatment according to hospital standard of care.

If you decide to take part, thank you! You will get a copy of this Information Sheet and Consent Form to keep.

What is the study about?
This study is trying to understand how sickness in babies works. Researchers want to compare healthy babies with sick babies to learn how to better treat or cure sickness in babies.

Why was my healthy baby and I chosen?
We are studying babies with a condition called 'NESHIE' (Neonatal Encephalopathy with Suspected Hypoxic Ischaemic Encephalopathy). This means a baby didn't get enough oxygen to their brain, usually

during birth, which can cause brain damage. This damage can lead to problems like movement issues, such as cerebral palsy. We also want to collect information and samples from babies who don't have NESHIE (like your baby). This will help us learn more about how sick babies are different from healthy ones. We will explain what we mean by "samples" in the next section.

What will happen to my baby and me, and what samples are being collected?

We want to gather information about you, your pregnancy, your labour, and your baby. We also want to collect blood from the umbilical cord and a pea-sized piece of tissue from the placenta. This information and these samples will help us better understand why some babies get sick and develop NESHIE.

Normally, the medical team will throw away the placenta, umbilical cord, and the blood from the umbilical cord after the birth, unless you ask to keep them. For this study, we've asked the medical team to keep these samples until we have a chance to ask for your permission to use them. The blood and placenta will be kept safely until we can speak to you and ask for your permission (informed consent) to use the samples.

What happens if I don't give permission?

If you don't give permission, the samples will be thrown away, just like usual. Collecting the samples won't cause any extra pain or harm to you or your baby.

What samples are being collected?

Below is a list of the samples. We'll explain why we need each one:

☐ **Umbilical cord blood to study your baby's genes**

Blood from the umbilical cord ("cord blood") is usually thrown away after birth. Taking this blood doesn't harm you or your baby. We will use it to learn about your baby's genes (called "genome") and how they work in the blood (called "epigenome" and "transcriptome").

☐ **Umbilical cord blood for blood spot test**

We took a small amount of blood from your baby's umbilical cord and put it on a card to study natural molecules (called "proteins" and "metabolites") in the blood. These are substances the body makes naturally. Proteins and metabolites tell us about the health of a body and how the body is working at the time when samples are collected.

☐ **Placenta (after birth) and placenta sample**

Normally, the placenta is thrown away after birth, unless it's needed for a special reason. Collecting the placenta and a small sample from it doesn't affect you or your baby. We want to study the health of the placenta and test for micro-organisms (like bacteria) in it. To study

the health of the placenta, we study the whole placenta. To test for micro-organisms, we take one sample, no bigger than a pea, from the whole placenta.

We will only use these samples if you give us permission. If you do not give us permission, the hospital will throw them away. You can choose to let us use all the samples listed above or you can choose which samples we may use.

Medical information from the hospital files

We also want to use some medical information from your and your baby's hospital records. This includes things like your age, weight, health during pregnancy, and any infections you might have had. We may ask for more details if needed. If you live in the Western Cape, we might also get some health information from the local Department of Health. If you take part, we may also ask for information from any samples sent to the NHLS. This will only be done as needed for this study.

What are genes and DNA?

"Genes" are present in all of us and are the same in all parts of our bodies. They are made of DNA, which is the material that carries our genetic information. We study genes to understand why people in the same family often share similarities, like height, and why families are different from one another. This information is passed from parents to children, and from one generation to the next through genes, which make up the "genome".

Genes can also interact with factors like our environment, lifestyle, and daily habits, which can influence how our body works. When we study how genes work with these factors, we look at the "transcriptome", which shows which genes are active at a given time. Sometimes, a baby's health is affected by their genes. In this study, we want to compare the genes of your baby (without NESHIE) to the genes of babies who have NESHIE to understand what genes might play a role in this sickness.

What will happen to these samples?

To protect your identity, we will remove personal information and use a study code to label the samples. This process is called 'anonymisation.' The samples will be sent to special laboratories to measure things like proteins, metabolites, micro-organisms (like bacteria), and genetic material ("genome", "epigenome" and "transcriptome") that is present in the sample.

The only information that will be given with these samples will be the sample codes, the date and time the sample was collected, the age of the sample, and the type of sample that was collected (e.g. dry blood spot). The samples will be sent either to laboratories in South Africa or internationally, following strict safety rules. These samples may be stored for up to five years before being used.

To study the health of the placenta, it will be sent to a specialised laboratory in South Africa. The blood and placental tissue samples will be kept in a "biobank," a secure place that stores samples safely for

long periods of time. Only a small number of researchers can access these samples, but they won't know who the samples belong to, keeping your information private. Any samples not used by the end of the study will be thrown away.

However, we may ask you if you would like to take part in other studies. If you agree to take part in other studies, we may keep the samples for later use. Should your samples be used for anything other than control samples, we will contact you again to get permission.

What will happen to the data from these samples?
All the information collected from you and your baby, along with the test results, will be stored in a secure database called the NESHIE database. A database is like a big digital folder where you can store lots of information in an organised way. Only the people involved in this study will be able to access it. The data might be used in scientific papers or presentations, but your identity will remain protected.

We will only use your baby's information and samples to compare with those of sick babies, we will not be studying your baby specifically. Because of this, we won't share the results with you or anyone you know. If you agree, we will use your baby's information and samples only for research and development.

Some anonymised information, such as the epigenomic, transcriptomic, proteomic, metabolomic, and microbiomic information may be shared with approved data repositories for research purposes. A data repository is like a big, organised storage space where lots of information is kept. No personal information that can identify you will be placed in the data repository.

How long will the samples and data be used?
The information and samples will be kept for at least 10 years after the study ends.

Are there any risks?
Collecting the samples, like umbilical cord blood and placental tissue, is harmless and will not cause any pain or risk to you or your baby.

Are there any benefits?
There are no direct benefits for you or your baby from this study right now because we are not testing any new treatments. However, what we learn from this study may help sick babies in the future. All babies, whether in the study or not, will receive the usual care in the hospital. If you join the study, the information and samples we collect will help doctors understand sick babies better and may lead to new ways to test and treat them. These new methods could help babies in South African public hospitals in the future, though it's not clear when that might happen. You may not see any benefit directly, but we hope the study helps improve care for sick babies later on.

Do I have to take part?

No, it is your choice whether you want to take part. Your and your baby's care will not be affected whether or not you decide to be part of the study. You and your baby will still receive the same medical care as all other mothers and babies in the hospital.

What if I change my mind?

You can change your mind at any time, even after you've agreed to be part of the study. You can choose to leave the study or stop certain parts of it without needing to explain why. If you decide to leave, you and your baby will still receive the same healthcare as before. The person running the study can also remove your baby from the study if it's in your baby's best interest.

If you want to withdraw, you can talk to your doctor or contact the people listed in the document. When your child turns 18, they can also choose to leave the study.

If you withdraw, we will erase your information and any unused samples. If some of your samples have already been used, we may not be able to remove shared information, but we will still destroy the remaining samples. Your data and samples will be removed from the storage so they can't be used in the future.

Will my information and my baby's information be kept confidential?

Your data will be collected, processed and stored according to the South African Protection of Personal Information (POPI) Act of 2013. We will keep all the information you share with us private. This means no one outside the study or your baby's healthcare team will see it. Some members of the research team who are not at the hospital may also need to access your information.

Your personal details, like names, will be replaced with a secret code to protect your privacy. When we share study findings, no information that could identify you will be included. All the information will be stored safely in a special computer database. Only people involved in the study can access it, unless we tell you otherwise. If the information is shared with other researchers, they will only see it in a way that keeps your identity hidden. Any information used in reports or publications will be anonymous.

Since each baby has a unique set of genes, we are extra careful with genetic samples and the information from them. Genetic samples will only be used in the way you agree to it being used.

Will my baby and I be paid?

There will be no payment for participating in the study. The study will not make money from your samples, and no payments will be made if products are developed from the study. Your samples or information will never be sold.

Will I be kept informed?

We would like to share our progress with you but will make sure your privacy is always protected. If you want, we can send updates about the study to you via SMS or email. To do this we will give you a form to complete so that we are able to contact you for this.

Are there other studies I could join?

At the end of this form, we will ask if we can use some of your and your baby's health information and samples for other studies related to NESHIE or newborn and maternal health in the future. Your samples will only be used as control samples. If we want to use them for anything else, we will ask you for permission first. You can choose whether or not to agree, and your decision will not affect your care or your participation in this study.

No data or samples can be used for research without your permission. If you agree to let us use them for future studies related to NESHIE or neonatal and maternal health, we must get approval from a Human Research Ethics Committee. If they approve, the samples and data will only be used for research and will follow the committee's rules.

Who reviewed this study?

The study has been approved by ethics committees at several universities and the National Health Research Database. This study follows ethical guidelines (Declaration of Helsinki (last update: October 2024 and SA GCP 2020 guidelines)) to protect participants, ensure ethical conduct and compliance with local regulations.

If at any time you would like to contact the [University specific REC name here] for questions on the ethics part of this study, you may contact:

REC Chair:	[University specific REC Chair name here]
E-mail:	[University specific REC contact details here]
Tel:	[University specific REC contact details here]

Who is funding this research?

The research is funded by the South African Medical Research Council and the Bill and Melinda Gates Foundation.

What if I have questions?

If you have any questions about the study, you can contact the study team member listed below for more information.

[Research member name] during office hours on [number] or e-mail at [email address].

PARENTAL PERMISSION FORM

& NEONATAL INFORMED CONSENT DOCUMENT

Before agreeing to take part, you should understand what is involved.

The information leaflet and consent form give information to help you decide if you and your baby would like to take part in this study.

In the information leaflet about this study, we have answered these questions:

- What is the study about?
- Why was my healthy baby and I chosen?
- What will happen to my baby and me, and what samples are being collected?
- What happens if I don't give permission?
- What samples are being collected?
- What are genes and DNA?
- What will happen to these samples?
- What will happen to the data from these samples?
- How long will the samples and data be used?
- Are there any risks?
- Are there any benefits?
- Do I have to take part?
- What if I change my mind?
- Will my information and my baby's information be kept confidential?
- Will I or my baby be paid?
- Will I be kept informed?
- Are there other studies I could join?
- Who reviewed this study?
- Who is funding this research?
- What if I have questions?

If you decide you want your baby to join the study, you'll be asked to sign the permission statement at the end of this form. You'll get a copy of the form to take home. Your baby won't be included in the study until you've read and signed the form. You can also talk to your partner, family, friends, or your baby's doctor about the study if you want. You and your baby can leave the study at any time, without needing to explain why. Deciding not to join or leaving the study won't affect the care you or your baby get at the hospital.

S2 File. Annexure 2: Informed Consent Documentation

Parental Permission and Informed Consent

I have received enough information in a way that I can understand clearly, in order to be able to decide for myself whether to take part in this study:

☐ **YES** ☐ **NO**

I agree to take part in the control part of the NESHIE study as it has been described to me. I understand that the results of the study, including my baby's medical information and my own will be put into a study report, and may be published. I also understand that my identity and the identity of my baby will be protected and kept secret.

☐ **YES** ☐ **NO**

I understand that if we take part in this study, or if we choose to not take part, my baby and I will still receive the same standard of care as all other healthy babies in the hospital:

☐ **YES** ☐ **NO**

I understand that I can decide to leave the study at any time, and if I leave the study my baby's medical care will not be affected by this decision:

☐ **YES** ☐ **NO**

If I decide to leave the study, I understand that all the unused samples and information from me and my baby will be disposed of wherever possible:

☐ **YES** ☐ **NO**

I understand that when my samples and medical information are collected, they will be kept and used as control information and data for the NESHIE study. I understand that the samples may be shipped to laboratories within or outside of South Africa. I understand that the researchers will adhere to all regulatory requirements for shipping and testing of samples:

☐ **YES** ☐ **NO**

I understand that systems/servers outside of South Africa may be used for the data collected as part of this study and that this will only be done as necessary to achieve the study aims:

☐ **YES** ☐ **NO**

I understand that I will not receive payment if I agree that my baby and I will participate in the study. I also understand that information and samples collected and generated as part of this study will never be sold:

☐ **YES** ☐ **NO**

I give permission for the following samples and medical information to be collected, kept and used as described:

**Sample type**	**Agree to sample collection, storage and use for the study**				**Patient initial**
Baby: Cord blood sample	Yes		No		
Baby: Blood spot card	Yes		No		
Baby: Medical information	Yes		No		
Baby: HIV status & treatment information	Yes		No		
Mother: Placenta tissue sample	Yes		No		
Mother: Whole placenta	Yes		No		
Mother: Medical information	Yes		No		
Mother: HIV status & treatment information	Yes		No		

If there are other, different studies about NESHIE in the future, our samples and information can be used in those NESHIE-related studies as control samples/data, subject to approval from the University of the Witwatersrand, Human Research Ethics Committee: (Medical).

☐ **YES** ☐ **NO**

By signing and dating this permission form, I have not waived any of the legal rights that I would normally have. I will receive and may keep a copy of this signed and dated permission form.

**MOTHER -** name and surname	Mother's signature	Date
**FATHER -** name and surname	Father's signature	Date
Investigator's name and surname	Investigator's signature	Date
Witness's name and surname	Witness's signature	Date

If both parents are not able to consent, the consenting parent must sign and date the clause below. *Please ignore if both parents were able to sign and consent.*

The other adult(s) with whom I share parental rights and responsibilities in respect of the identified child in terms of the Children's Act (for example, biological parent, adoptive parent, or legal guardian or representative) is:

(1) aware of and agrees with my granting permission for this child to participate in the study

OR

(2) deceased, unknown, incompetent, or not reasonably available (someone is "not reasonably available" when he/she cannot be reached by phone/mail/email/text because, for example, he/she is on active military duty or is incarcerated).

Name and surname of consenting parent	Signature	Date

**<u>AUTHORISED PERSONNEL TO COMPLETE</u>**	
Mother hospital number:	
Name & surname of person performing recontact -consent:	
Signature:	
Signature date:	
Was a signed copy of this document given to the parent(s)	Yes / No

Annexure 2.3: Informed Consent Checklist

NESHIE COMPARATIVE STUDY

INFORMED CONSENT CHECKLIST AND PROCESS

Study ID:	HEALTHY CONTROLS

FOR ALL QUESTIONS: ENSURE THAT THE APPROPRIATE BOX IS MARKED WITH AN 'X'

Question	Document	Response	If NO
. Did a research team member speak to the parent(s) about the control arm NESHIE study?		YES / NO	→ **ONLY CONSENT AFTER BEING INFORMED**
. Did the mom sign a recontact form?		YES / NO	→ **COMPLETE 'YES' OR 'NO' & PROCEED TO Q3**
. If **Q2** is **'YES'**, is the mother of the neonate at least 18 years of age?		YES / NO	→ **DO NOT CONSENT**
. If **Q3** is **'YES'**, were the following documents given to the parent(s)?	1. Study Information Leaflet	YES / NO	
	2. Neonatal ICF	YES / NO	
. If **CONSENT** was **OBTAINED**, were the following documents given to the parent(s)?	1. Community Engagement Form	YES / NO / Not interested	
. If consent was obtained, were **signed copies** of the following documents given to the parent(s)?	1. Neonatal ICF	YES / NO	
	2. Community Engagement	YES / NO	
. I/We can confirm that **scanned copies** of all **signed consent** documents have been made.		YES / NO	→ **SCAN ALL SIGNED CONSENT FORMS**
