# Supplementary material for: Protocol: A multi-factorial, multi-centre study, for biomarker identification in healthy controls for comparison to babies with moderate-severe NESHIE
Source: PLoS One. 2026 Apr 8;21(4):e0346798. doi: 10.1371/journal.pone.0346798 (PMC13061247; doi:10.1371/journal.pone.0346798)
Supplement: S3 File — Standardised data capture forms used to record maternal and infant clinical data. (PDF) [file pone.0346798.s003.pdf]

Annexure 3: Data Capture Forms for NESHIE Healthy Controls

Study ID Control Neonate:

NESHIE COMPARATIVE STUDY: MASTERLIST DETAILS HEALTHY CONTROLS

(Keep separate from Neonatal and Maternal CRFs & retained at site-level only)

**NEONATAL STUDY ID**	
**Surname**	
**Infant hospital folder no**	
**CLS CP number**	

**MATERNAL STUDY ID**	
**Maternal DOB**	
**Maternal hospital folder no**	
**CLS CP number**	

Data highlighted in yellow will be captured to the CRF but not to the electronic database. This will be done to be able to retrace the hospital record if ever necessary, but also protect the participant’s identity.

NESHIE COMPARATIVE STUDY: INCLUSION/EXCLUSION CRITERIA (HEALTHY CONTROLS)

**COMPLETE NESHIE SCREENING FORM if category A & B conditions are met.**<br>**FOR ENROLMENT INTO THE NESHIE STUDY: All inclusion criteria should be met with no exclusion criteria present**

**A: Gestation and weight criteria (All must be 'Yes' to be included in study)**		
≥ 36 weeks gestation*	**Yes**	**No**
≥ 1800g	**Yes**	**No**

* As determined by foetal ultra sound at ≤ 20 weeks; or postnatal Ballard, or Foot length measurements (≥73mm in length)

**B: Health criteria (All must be 'No' to be included in study)**		
Were any clinical signs of intrapartum hypoxia/birth asphyxia noted?	**Yes**	**No**
Were any clinical signs of neonatal encephalopathy noted?	**Yes**	**No**

**C: Exclusion criteria**<br>(Tick all that apply. The presence of any exclusion criteria disqualifies the patient from enrolment.)					
None		Mother underage (<18 years of age)		Neonate considered for, or received cooling	
Required assisted ventilation (IPPV) after birth		Suspected chromosomal abnormality		Congenital infection	
Consent refused		Neonate died prior to obtaining consent			
Consent not obtained for other reason	State:				
Congenital abnormality	State:				
Other	State:				

**D: Can it be confirmed that the parent(s) provided informed consent?**<br>(Must be "YES" to be included in the study)	Yes	No

**E: Was the patient recruited?**	Yes	No
If "no", why not?		

NESHIE COMPARATIVE STUDY: SCREENING SHEET (HEALTHY CONTROLS)

NEONATAL SCREENING INFORMATION									
**Hospital**									
**DOB**				**TOB**					
**The neonate was:**	Inborn		Outborn		**Sex**	M	F	Intersex	

BIRTH SITE INFORMATION			
**Province**			
**Health Sub-district**			
**Name of Institution**			
**Is the Province in which the mother gave birth the same as the province in which the mother permanently resides?**		Yes	No
**If no, in which Province does the mother reside permanently?**			

NESHIE COMPARATIVE STUDY: SAMPLE COLLECTION SHEET (HEALTHY CONTROLS)

Fill in details or check the applicable answer

**PLACENTA SENT FOR HISTOLOGY/PATHOLOGY**	Yes	Date sent:	No	N/A
If the placenta was sent for histology, please ensure that all lab details are captured and stored to the site master-list. Complete the placental pathology section once the completed report has been received				

**Placenta: Pathomicrobiome sample collected?**		[1] The date and time of sample collected should be captured directly from the requisition form into REDCap				Yes[1]	No	N/A
If 'No'	Explain why not collected:		Consent refused		Placenta discarded	Other:		

**Cord blood (Venous): DNA & RNA sample collected?**						Yes[1]	No	N/A
If 'No'	Explain why not collected:		Consent refused		Cord discarded	Other:		
If 'Yes'	Date & Time Collected: [1] The date and time of sample collected should be captured directly from the requisition form into REDCap	Source of sample	Cord: Venous		Cord: Arterial		Cord: Mixed	
		Anticoagulant?[2]	None		EDTA		Other	
			List '**Other**' anticoagulant:					

**Blood spot: 0-6 hour sample collected?**						Yes	No	N/A
If 'No'	Explain why not collected:		Consent refused		Cord discarded	Other:		
If 'Yes'	Date & Time Collected: [1] The date and time of sample collected should be captured directly from the requisition form into REDCap	Source of sample	Cord: Venous		Cord: Arterial		Cord: Mixed	
		Anticoagulant?	None		Heparin		Other	
			List '**Other**' anticoagulant:					

NESHIE COMPARATIVE STUDY: ASSESSMENT AT BIRTH (HEALTHY CONTROLS)

**CLINICAL DETAILS OF BABY AT BIRTH**<br>*Check relevant block and provide details as requested and as applicable*									
**Method used for GA**	Dates		EUS at ≤20 weeks		**Values for GA method**		Ballard score		
	Ballard		Foot length				Foot length		mm
**Apgar scores:**	1 min		5 min		10 min		**Body Length**		cm
**Gestation at birth**		wks	**Birth weight**			g	**COH**		cm

**WERE ANY OF THESE PRACTICES OBSERVED OR RECORDED ON FILE?**								
**Adrenaline**	Yes	No	**BMV**	Yes	No	**Chest compressions**	Yes	No
**Delayed cord clamping / Cord milking**	Yes	No	**Intubated**	Yes	No	**Blood gas results done and available**[1]	Yes	No

[1] Worst base excess within 60 minutes of birth including cord blood

**EVALUATIONS: AT BIRTH (IF DONE)**<br>*Provide details as requested and as applicable*								
**Blood glucose**		mmol/L	**POC: Potassium (K)**		mmol/L	**pH** (POC)		
**POC: Sodium (Na)**		mmol/L	**POC: Base Excess**		mmol/L	**POC: Bicarb**		mmol/L
**POC: Lactate**		mmol/L	**PaO₂**........................	kPa	mmHg	**POC: iCa**		mmol/L

**PCO_2...............(POC)**	kPa	mmHg					
**Lab: Hb**		g/dL	**Lab: NRBC**		%	**Lab: WBC**		cells/L
**Lab: Neutrophils**		cells/L	**Lab: Platelets**		cells/L			

**NEUROLOGICAL ASSESSMENT AT OR AROUND TIME OF BIRTH (WITHIN 6 HOURS OF LIFE)**						
**Date taken**		**Time taken**		**Age at neurological assessment**	hr	min
**Thompson HIE score** [1]		**Modified Sarnat HIE grade**		**Visible seizures**	Yes	No

[1]Please capture the clinical features associated with this score in the table below

**THOMPSON HIE SCORE FEATURES**	**Tone**		**LOC**		**Visible fits**		**Posture**		**Moro**	
*Please circle the number linked to each of the Thompson HIE score observations reflected by the total score captured in the **neurological assessment** block.*	0	Normal	0	Normal	0	None	0	Norm/Other	0	Norm
	1	HypErtonic	1	HypErtonic	1	Infrequent < 3/day	1	Fisting and/or Cycling	1	Partial
	2	HypOtonic	2	HypOtonic	2	Frequent > 2/day	2	Strong distal flexion	2	Absent
	3	Flaccid	3	Flaccid			3	Decerebrate		
	**Grasp**		**Suck**		**Respiration**		**Fontanel**		**TOTAL**	
	0	Norm	0	Norm	0	Norm	0	Norm		
	1	Poor	1	Poor	1	Hyperventilation	1	Full		
	2	Absent	2	Absent	2	Transient apnoea	2	Tense		
					3	Apnoea requiring IPPV				

**CLINICAL EXAMINATION FROM TIME OF BIRTH TO DISCHARGE**								
Check relevant block if noted upon examination								
**CPAP**	Yes	No	**Nasal Cannulae**	Yes	No	**Additional oxygen provided? (E.g. FiO_2>21%)**	Yes	No
**Mechanical ventilation**	Yes	No	**Hypotension or Inotropes**[1]	Yes	No	**Active bleeding treated with blood product**	Yes	No
**Subaponeurotic haemorrhage**	Yes	No	**Sinus bradycardia <80**	Yes	No	**Arrhythmia (other than SB <80)**	Yes	No
**Necrotising enterocolitis**	Yes	No	**Late onset sepsis (> 72h)**	Yes	No	**Meconium aspiration**	Yes	No
**Death**	Yes	No	**None of these**	Yes	No			

[1] Hypotension = MBP persistently < 40mmHg

NESHIE COMPARATIVE STUDY: MATERNAL & OBSTETRIC DATA (HEALTHY CONTROL)

NOTE: IF A TEST / EVALUATION WAS NOT DONE, PLEASE INDICATE THIS USING "ND". IF A TEST / EVALUATION WAS DONE BUT DATA / RESULTS ARE NOT KNOWN, PLEASE INDICATE THIS USING "UK"

**MATERNAL DEMOGRAPHIC DETAILS**<br>*Provide details as requested and as applicable*	
**First language**	

**MATERNAL PREGNANCY DETAILS**<br>*Check relevant block and provide details as requested and as applicable*									
**Confidence in date of last menstrual period**	Sure		Unsure		**Did the mother receive antenatal care before deliver (was she "booked")?**	Yes [1]		No	
**Date of last menstrual period**					**Date of 1st ultrasound scan**				
**Gestational age by dates**	weeks				**Gestational age at 1st ultrasound scan**	weeks			
**Estimated date of delivery by: Dates**					**Estimated date of delivery by: 1st ultrasound**				
**Was weight measured at 1st booking?**	Yes	No	Unknown		***If " Yes"*** **Weight** kg	**Height** cm			

**Hospital where birth took place**				**Age at time of delivery**	years		
**Gravidity**		**Parity**		**How many foetuses this pregnancy?**		**No. previous miscarriages (<22w)**	
				**No. previous stillbirths (≥22w)**			
[1&23] Please complete Tables 1-3 as appropriate				**No. previous newborns admitted?** [1]		**No. previous newborns with NESHIE / 'Asphyxia'?** [2]	
**Postnatal measurements:**	Not done			**M/U arm circ** [3] cm	**Weight** kg	**Height** cm	
**Lowest antenatal Hb** (g/dL)							

**RVD / HIV status**[4]	Positive + ARV		Positive No ARV		Negative		Unknown	
**Venereal disease status**	Positive Fully treated		Positive Not fully treated		Negative		Unknown	
**Venereal disease test(s) used**	VDRL		RPR		TPHA			

[1] **Please complete Table 1 if previous newborns were admitted,**
[2] **Please complete Table 2 if there had previously been NESHIE / 'Asphyxia' cases**
[3] **Measurements taken on RIGHT arm**
[4] **Complete Table 3 if mother is HIV-positive**

COMPLETE THE INFORMATION IN TABLE 1 ONLY IF: PREVIOUS NEWBORNS HAD BEEN ADMITTED
COMPLETE THE INFORMATION IN TABLE 2 ONLY IF: THERE WERE PREVIOUS NESHIE / 'ASPHYXIA' CASES
COMPLETE THE INFORMATION IN TABLE 3 ONLY IF: THE MOTHER TESTS POSITIVE FOR HIV

Table 1: Birthweight and reason for previous admissions

Admission number	Birthweight (grams)	Reason for admission	Did the newborn demise?		
1			Yes	No	Unknown
2			Yes	No	Unknown
3			Yes	No	Unknown

Table 2: Long-term problems for previous NESHIE / 'Asphyxia' newborns

Admission number	Long-term problem(s)	Did the newborn demise?		
1		Yes	No	Unknown
2		Yes	No	Unknown
3		Yes	No	Unknown

Table 3: Details pertaining to HIV status*

**If HIV positive, last CD4 count**	cells/µL	CD4 unknown		**Date of test**	
**Viral load count: At first 'booking'**	copies/mL	VL unknown		**Date of test**	
**Viral load count: During pregnancy**	copies/mL	VL unknown		**Date of test**	
**Viral load count: During pregnancy**	copies/mL	VL unknown		**Date of test**	
**Viral load count: During pregnancy**	copies/mL	VL unknown		**Date of test**	
**Viral load count: At birth (mother)**	copies/mL	VL unknown		**Date of test**	
**Viral load count: At birth (neonate)**	copies/mL	VL unknown		**Date of test**	

*** If more than four VL counts are taken during pregnancy, please record this information separately, capture to the electronic database and upload alongside the completed CRF.**

**MATERNAL MEDICAL CONDITIONS OR TREATMENT PRESENT PRIOR TO PREGNANCY? (NOT PREGNANCY COMPLICATIONS)**									Yes	No	UK
Check relevant block and provide details as requested and as applicable below if "Pregnancy complications" is "Yes"											
**Anaemia**	Yes	No	UK	**Cardiac disease**	Yes	No	UK	**Diabetes**	Yes	No	UK
**Epilepsy / Seizure**	Yes	No	UK	**Hypertension**	Yes	No	UK	**Thyroid disease: Hypothyroidism**	Yes	No	UK
**Thyroid disease: Hyperthyroidism**	Yes	No	UK	**Other**	Yes	No		**Detail if "Other" is "Yes"** ………………………………			
………………………………………………………………………………………………											
………………………………………………………………………………………………											
**Treatment for pre-existing conditions?**	Yes	No	UK	**Detail if "Yes"** ……………………………………………………………							
………………………………………………………………………………………………											
………………………………………………………………………………………………											

COMPLICATIONS DURING PREGNANCY BEFORE ONSET OF LABOUR / PREGNANCY COMPLICATIONS?									Yes	No	UK
Check relevant block and provide details as requested and as applicable below if "Pregnancy complications" is "Yes"											
**Anaemia (gestational)**	Yes	No	UK	**APH / Bleeding**	Yes	No	UK	**Clinical Chorioamnionitis**	Yes	No	UK
**Diabetes (gestational)**	Yes	No	UK	**Hypertension (gestational)**	Yes	No	UK	**Intra-uterine growth restriction**	Yes	No	UK
**Placenta Praevia**	Yes	No	UK	**Pre-eclampsia / eclampsia / HELLP**	Yes	No	UK	**PROM > 18h**	Yes	No	UK
**Pyrexia**	Yes	No	UK	**TB (Active) during pregnancy**	Yes	No	UK	**On treatment if "TB"= "Yes"?**	Yes	No	UK
**Other**	Yes	No		**Detail if "Other" is "Yes"** …………							
…………											
…………											
**Infection during pregnancy?**	Yes	No	UK	**Selection option only if "Infection" is "Yes"**	Urinary			Vaginal/Vulval		Other	
**Detail if "Infection" is "Other"**											

RECREATION / HABITS DURING PREGNANCY?				Yes	No	UK
Check relevant block and provide details as requested and as applicable if "Yes" to any recreational habits						
**Alcohol**	Yes	No	UK	Description:		
**Cigarettes / Smoker**	Yes	No	UK	Description:		
**Illicit drugs**	Yes	No	UK	Description:		
**Other**	Yes	No	UK	Description:		

MEDICATIONS (NOT SUPPLEMENTS) USED IN PREGNANCY?	Yes	No	UK
If applicable, please capture details of medications used in **Table 4**			

CTG DATA BEFORE ONSET OF LABOUR CONTRACTIONS (<24 HOURS BEFORE ONSET OF LABOUR)								
Scan last 30 minutes of CTG before onset of labour if available								
**CTG <24 hours before onset of labour**	Available		Not done		Done, not available			
**Reason for CTG done (main risk factor):** ………… …………								
**COMPLETE LINE BELOW ONLY <u>IF NO CTG</u> AVAILABLE / DONE <24 HOURS BEFORE ONSET OF LABOUR**								
**If "no CTG", was FHR normal/reactive or reassuring?**	Yes	No	Unknown	**Decelerations noted?**	Yes	No	Unknown	

ONSET OF LABOUR						
Check relevant block and provide details as requested and as applicable						
**Onset of labour**: Admission or first noted while admitted	**Date**		**Time**			
**Nature of labour**	Spontaneous		Induced		Caesarean Section (not in labour)	
**Are there other notes on the nature of labour?**	Yes	No	Describe if "Yes" …………			
…………						
**Method of induction**	Catheter bulb		PGE2		Misoprostol	

**(Check all that are applicable)**	Oxytocin		AROM		Other	
**Describe if "induction" is "Other"**						
**Fetal movements at onset of labour**	Normal		Reduced		Not noted	

**FETAL CONDITION AFTER ONSET OF LABOUR CONTRACTIONS**								
Scan all available CTG trace after onset of labour								
**CTG in labour earlier than the last 2 hours before delivery**	Available		Not done		Done, not available			
**COMPLETE LINE BELOW ONLY <u>IF NO CTG</u> AVAILABLE / DONE AFTER ONSET OF LABOUR**								
**If "no CTG", was FHR normal/reactive or reassuring?**	Yes	No	Unknown	**Decelerations noted?**	Yes	No	Unknown	

**MATERNAL CONDITION AFTER ONSET OF LABOUR CONTRACTIONS**								
**Highest maternal heart rate in last four hours of labour**	<100 bpm		101-110 bpm		111-120 bpm		> 120 bpm	
**Analgesia in labour** (Check all that are applicable)	None		Opiate		Epidural		Other	
**Describe if "Analgesia" is "Other"**								
**Duration of oxytocin augmentation (if used; completed hours)**	N/A	hours	**Highest temperature in labour**	°C				
**Duration of active phase of first stage**	1st time found cervix ≥ **4 cm** to 1st time 10 cm	h min	1st time found cervix ≥ **5 cm** to 1st time 10 cm	h min				
**Duration of second stage**	1st time found cervix 10 cm to birth	min	**Duration of ruptured membranes**	hours				
**Liquor**	Clear		Mec+		Mec++		Mec+++	
**Offensive liquor**	Yes	No						

**LAST 2 HOURS OF FHR/CTG IN LABOUR**							
Scan all CTG tracing in the last 2 hours before delivery							
**CTG from last 2 hours before delivery**	Available		Not done		Done, not available		
**COMPLETE LINE BELOW ONLY <u>IF NO CTG</u> AVAILABLE FOR >30 MINS AND DONE IN LAST 2 HOURS OF LABOUR**							
**If "no CTG", was FHR normal/reactive or reassuring?**	Yes	No	Unknown	**Decelerations noted?**	Yes	No	Unknown

**DELIVERY COMPLICATIONS / SENTINEL EVENTS?**											Yes	No	UK
Complete block below only if "Delivery Complications" is "Yes"													
**Fetomaternal haemorrhage**	Yes	No	UK	**Other Ante Partum Haemorrhage**	Yes	No	UK	**Placental abruption**	Yes	No	UK		
**Maternal Hypoxia**	Yes	No	UK	**Maternal collapse**	Yes	No	UK	**Sudden onset bradycardia**	Yes	No	UK		
**Prolapse Cord**	Yes	No	UK	**Ruptured Uterus**	Yes	No	UK	**Prolonged 2nd Stage (>2 hours)**	Yes	No	UK		
**Shoulder Dystocia**	Yes	No	UK	**Difficult breech**	Yes	No	UK	**Other Sentinel Event(s)**	Yes	No	UK		

Detail if "Other Sentinel events" is "YES": ....

....

**UMBILICAL CORD ABNORMALITIES** *(Check relevant block)*	None		True knot		Cord around neck twice or more		Single artery	
	Other		Abnormal insertion		Hypercoiling		Unknown	
	Detail “Other” or “Abnormal insertion”………………………………………………………………………………………………… ………							

**PLACENTAL ABNORMALITIES** *(Check relevant block)*	None		Abnormal		Unknown		
	Detail if “Abnormal” …… ………						

**ROUTE OF DELIVERY** *(Check relevant block)*	Pre-labour CS		In-labour CS		Vaginal	
**PRESENTATION**	Cephalic		Breech			
**MODE OF (VAGINAL) DELIVERY** *(Check relevant block)*	Spontaneous		Vacuum		**FAILED VACUUM OR FORCEPS?**	Yes
	Caesarean section		Forceps			No
Reason for vacuum or forceps …… ………						
Reason for caesarean section …… ………						

**DIFFICULTIES WITH DELIVERY**	Yes	No	UK
If yes, detail ……… ………			

Table 4: Medications used during pregnancy

MEDICATION DETAILS: ***Complete only if the mother received and used medication during pregnancy***

**Drug generic name**								
**Chronic or Acute medication?**	Chronic		**Dosage**			**Dosage Unit**	mg	µg
	Acute		**Frequency**	1 x Daily		2 x Daily		
**Duration of use**		D/M/Y		3 x Daily		Other		
Any other details noted RE Medication: …………………………………………………………………………								

**Drug generic name**								
**Chronic or Acute medication?**	Chronic		**Dosage**			**Dosage Unit**	mg	µg
	Acute		**Frequency**	1 x Daily		2 x Daily		
**Duration of use**		D/M/Y		3 x Daily		Other		
Any other details noted RE Medication: …………………………………………………………………………								

**Drug generic name**								
**Chronic or Acute medication?**	Chronic		**Dosage**			**Dosage Unit**	mg	µg
	Acute		**Frequency**	1 x Daily		2 x Daily		
**Duration of use**		D/M/Y		3 x Daily		Other		
Any other details noted RE Medication: …………………………………………………………………………								

**Drug generic name**								
**Chronic or Acute medication?**	Chronic		**Dosage**			**Dosage Unit**	mg	µg
	Acute		**Frequency**	1 x Daily		2 x Daily		
**Duration of use**		D/M/Y		3 x Daily		Other		
Any other details noted RE Medication: …………………………………………………………………………								

**Drug generic name**								
**Chronic or Acute medication?**	Chronic		**Dosage**			**Dosage Unit**	mg	µg
	Acute		**Frequency**	1 x Daily		2 x Daily		
**Duration of use**		D/M/Y		3 x Daily		Other		
Any other details noted RE Medication: …………………………………………………………………………								

* D/M/Y indicates use over **D**ays, **M**onths, or **Y**ears
