# Supplementary material for: Protocol: A multi-factorial, multi-centre study, for biomarker identification in healthy controls for comparison to babies with moderate-severe NESHIE
Source: PLoS One. 2026 Apr 8;21(4):e0346798. doi: 10.1371/journal.pone.0346798 (PMC13061247; doi:10.1371/journal.pone.0346798)
Supplement: S4 File — Document used to guide and record community engagement activities related to the study. (PDF) [file pone.0346798.s004.pdf]

S4 File. Annexure 4: Community Engagement Form

Study number:________________ Annexure 4

NESHIE Comparative Study Community Engagement Contact Form

Although we will keep your identity a secret, we would like to share our progress in the project with you. If you would like us to share this information with you directly, please give us your contact details on this form. You will not be forced to do this. You are free to choose if you would like to receive this information. We will share this information with you once every 6-12 months for as long as the study continues. This study will last for at least five years, but may continue over a longer period. We will keep your contact information private and confidential and we will not share it in any way. You may choose to stop receiving this information at any time. If your contact details change and you would still like us to share this information with you, you will need to inform us of these changes.

I have been made aware of the fact that healthcare professionals and researchers would like to keep me informed on the progress of the above-mentioned project that I have freely chosen to take part in. I have also been made aware that this information will be treated confidentially and will not be given out to any person who is not a member of the medical and research team involved in this project. I understand that I will, at most, receive updates once every 6-12 months over a five year period. I also understand that I can change my mind and choose not to receive updates at any point in time during the study and will share this decision with my doctor. I would like to receive information through one or more of the following options:

**Contact option**	**Please circle your choice(s)**	
Newsletter (via e-mail)	Yes	No
Update via SMS	Yes	No

Please provide the following information (if willing and available):

Cell number: ____

e-mail address: ____

Signature: ______________________________ Date: __________________
