# Supplementary material for: Protocol: A multi-factorial, multi-centre study, for biomarker identification in healthy controls for comparison to babies with moderate-severe NESHIE
Source: PLoS One. 2026 Apr 8;21(4):e0346798. doi: 10.1371/journal.pone.0346798 (PMC13061247; doi:10.1371/journal.pone.0346798)
Supplement: S5 File — Details of sample size calculations performed for the study. (PDF) [file pone.0346798.s005.pdf]

Annexure 5: Sample Size Calculation

Results from OpenEpi, Version 3, open source calculator--SSCC

Sample Size for Unmatched Case-Control Study

For:

Two-sided confidence level(1-alpha)	95
Power(% chance of detecting)	80
Ratio of Controls to Cases	499
Hypothetical proportion of controls with exposure	0.1
Hypothetical proportion of cases with exposure:	99.9
Least extreme Odds Ratio to be detected:	998001.00

	**Kelsey**	**Fleiss**	**Fleiss with CC**
Sample Size - Cases	1	1	1
Sample Size - Controls	12	9	303
Total sample size:	13	10	304

References

Kelsey et al., Methods in Observational Epidemiology 2nd Edition, Table 12-15
Fleiss, Statistical Methods for Rates and Proportions, formulas 3.18 &3.19

CC = continuity correction
Results are rounded up to the nearest integer.
